# Responsibility of the Insane

**Published:** 1898-01-22

**Authors:** 


### The Hospital

A Journal of

The Medical Sciences and Hospital Administration.

Vol. XXIII.—No. 591.] Edited by Sir Henry Burdett, K.C.B., and Solomon C. Smith, M.D., M.R.C.P. [Jan. 22, 1898.

### Responsibility of the Insane.

The escape of Prince, the murderer of Mr. Terriss, from capital punishment, and his condemnation in lieu thereof to the mere incarceration to which he ought to have been subjected long ago, cannot fail to direct the serious attention of the community, and especially that of neurologists, to some of the practical results of the deliverance to which the jury were guided by the evidence of experts, for it is hardly too much to say that this verdict has rendered the life of every British subject somewhat less safe than it was before.

Everyone conversant with insanity is well aware that the sufferers from that condition, at least in many of its forms and phases, are much more capable of self-control than is commonly supposed. If this were not the case the degree of liberty now accorded in asylums would be a source of constant danger both to inmates and to attendants. As a matter of fact, the inmates are quite amenable to discipline. They know quite well that acts of insubordination will be likely to entail discomfort of some kind upon those who commit them; and, because they know this, they yield, perhaps an imperfect and unwilling, but still a fairly effective, obedience to rules. It may be true that they possess self-control in only a feeble and diminished form, but so also do many people to whose insanity no doctor would venture to certify.

It may well be that a man who could not be called insane, but merely, say, an ordinary member of the criminal classes, with strong passions and feeble intellect, would, in the presence of an opportunity long expected and hoped for, the consequences of which his thoughts had frequently rehearsed, be really for the moment incapable of restraining his hand. We should hang him, nevertheless, without the slightest scruple; and we should waste no compassion upon the absence of self-control. It is, in fact, impossible to frame a definition of irresponsibility based upon absence of self-control without including all sorts of crimes which at present are punished by the law. For years back there has been a sort of feud between the lawyers and the alienists on the subject, so that even the text books speak of legal insanity and medical insanity as distinct. To take extreme cases, jurists have contended that no degree of insanity should exempt from punishment for crime unless it has reached such a point that the person is utterly unconscious of the difference between right and wrong at the time of committing the offence, while medical men have very generally held to the opinion that this is not a proper criterion, that many of the insane are fully conscious of the difference between right and wrong, and that to enforce such a test means the hanging of many a lunatic. There can be no doubt that of late years the medical view has met with a wider acceptance than it used to do, and that even lawyers have shown an increasing readiness to admit the doctrine of irresponsibility. But it is a very anxious question, especially in view of recent dogmas as to degeneracy, how far this doctrine is to be allowed to go.

The real question in this case then was not whether the prisoner was insane, but whether he was "insane so as not to be responsible for his actions." But this question the jury evaded, throwing all the onus on the medical evidence. The verdict was, "We say that he knew what he was doing and to whom he was doing it, but, on the medical evidence, that he was not responsible for his actions;" and, when specially asked the question whether he was "insane so as not to be responsible at law," the Foreman again hung on to what the medical witnesses had said, instead of giving a verdict according to the whole evidence, answering, "According to the medical evidence, yes." On this the prisoner was at once ordered to be detained as a criminal lunatic during Her Majesty's pleasure.

We do not here say anything against the verdict. Nevertheless we do feel bound to say that there are many lunatics now at large whose angry passions are restrained, in large degree, by exactly the same means as are found effectual in the case of their saner brethren—namely, the fear of punishment—and that it behoves us to be very careful not to remove such restraint by light sentences, unless we are prepared to extend very considerably such power as we may possess of shutting up angry and excitable persons who go about the world nursing their grievances—potential murderers.

The condition of affairs is much more serious than some people think, and it is highly necessary that those who administer the criminal law should be on their guard against any insidious establishment of immunity for the violation of its most sacred principles. In any case of murder, the presumption in favour of hanging should be so strong as to leave very slender prospect of escape for any man who, prior to the commission of his crime, had been thought fit to be at large. If this condition cannot be secured, it will become a matter of grave necessity to take prompt steps for the incarceration of many people of evil passions who are now at liberty, and to render the utterance of threats a matter to be dealt with by the alienist as well as by the magistrate.

